# Isolation of Tacaribe Virus, a Caribbean Arenavirus, from Host-Seeking *Amblyomma americanum* Ticks in Florida

**DOI:** 10.1371/journal.pone.0115769

**Published:** 2014-12-23

**Authors:** Katherine A. Sayler, Anthony F. Barbet, Casey Chamberlain, William L. Clapp, Rick Alleman, Julia C. Loeb, John A. Lednicky

**Affiliations:** 1 Department of Physiological Sciences, College of Veterinary Medicine, University of Florida, Gainesville, Florida, United States of America; 2 Department of Infectious Diseases and Pathology, College of Veterinary Medicine, University of Florida, Gainesville, Florida, United States of America; 3 Department of Pathology, College of Medicine, University of Florida, Gainesville, Florida, United States of America; 4 Department of Environmental and Global Health, College of Public Health and Health Professions, University of Florida, Gainesville, Florida, United States of America; 5 Emerging Pathogens Institute, University of Florida, Gainesville, Florida, United States of America; Division of Clinical Research, United States of America

## Abstract

*Arenaviridae* are a family of single stranded RNA viruses of mammals and boid snakes. Twenty-nine distinct mammalian arenaviruses have been identified, many of which cause severe hemorrhagic disease in humans, particularly in parts of sub-Saharan Africa, and in Central and South America. Humans typically become infected with an arenavirus through contact with excreta from infected rodents. Tacaribe virus (TCRV) is an arenavirus that was first isolated from bats and mosquitoes during a rabies surveillance survey conducted in Trinidad from 1956 to 1958. Tacaribe virus is unusual because it has never been associated with a rodent host and since that one time isolation, the virus has not been isolated from any vertebrate or invertebrate hosts. We report the re-isolation of the virus from a pool of 100 host-seeking *Amblyomma americanum* (lone star ticks) collected in a Florida state park in 2012. TCRV was isolated in two cell lines and its complete genome was sequenced. The tick-derived isolate is nearly identical to the only remaining isolate from Trinidad (TRVL-11573), with 99.6% nucleotide identity across the genome. A quantitative RT-PCR assay was developed to test for viral RNA in host-seeking ticks collected from 3 Florida state parks. Virus RNA was detected in 56/500 (11.2%) of surveyed ticks. As this virus was isolated from ticks that parasitize humans, the ability of the tick to transmit the virus to people should be evaluated. Furthermore, reservoir hosts for the virus need to be identified in order to develop risk assessment models of human infection.

## Introduction

*Arenaviridae* are a family of rodent and boid snake-associated, single stranded RNA viruses that currently (as of December, 2014) includes only one genus, *Arenavirus*. Twenty-nine distinct arenaviruses have been identified, many of which cause severe hemorrhagic fever (HF) in humans [1]. At least ten of these viruses are associated with human disease in many parts of the world including western Africa, Argentina, Bolivia, Venezuela and Brazil [2]. Specifically, areanviruses have caused significant mortality in humid pampas of central Argentina, and multiple Old World arenaviruses cause morbidity in endemic foci in sub-Saharan Africa [3].

These viruses are divided into two serogroups based on shared antigens and geographic distribution:(a) Lymphocytic Choriomeningitis-Lassa virus (LCM-LAS) serocomplex viruses, or the Old World arenaviruses, and (b) Tacaribe serocomplex viruses, or the New World arenaviruses, (NWV) [4]. Though LCM virus has a world-wide distribution, viruses of the LCM-LAS complex are found primarily in Africa, whereas the Tacaribe complex viruses are found in North and South America. The Tacaribe complex includes the following viruses: Tacaribe (TCR), Allpahuayo (ALL), Amapari (AMA), Bear Canyon Virus (BC), Chapare (CHP), Cupixi (CPX), Flexal (FLE), Guanarito (GTO), Junin (JUN), Latino (LAT), Machupo (MAC), Oliveros (OLV), Paraná (PAR), Pichinde (PIC), Pirital (PIR), Sabiá (SAB), Tamiami (TAM), and Whitewater Arroyo (WWA). Heretofore, the only NWV recognized by the International Committee on the Taxonomy of Viruses to occur in North America were the TAM, BC, and WWA viruses. The NWV are further subdivided into phylogenetic clades: A, B and C, and a recombinant clade. Although TCRV has been associated with one non-fatal laboratory acquired infection (mentioned briefly in reference 14), it is grouped in Clade B with the most pathogenic viruses, such as JUNV and MACV [5].

Arenavirus genomes are fairly simple, consisting of two ssRNA segments which encode four genes [6]. The large (L) segment contains genes for the RNA-dependent RNA polymerase (L protein) and the small RING-domain containing Z protein. The small (S) genome segment contains genes for the glycoprotein precursor (GPC) and the nucleoprotein (NP) of the virus. The placement of TCRV within phylogenetic clade B is based on the sequences of its GPC and NP genes, which encode proteins that are involved in host cell invasion [2], [4]. TCRV has been associated with a non-fatal, febrile laboratory acquired infections of humans, but is most closely related to JUNV, which is associated with up to 30% mortality in infected patients [7]. The virus is an important model organism for highly pathogenic Clade B viruses and has been investigated as a vaccine candidate for Argentine hemorrhagic fever [8]–[10]. In addition, TCRV provides an excellent model system for identifying the determinants of pathogenesis of the lethal arenaviruses, without the need for BSL-4 containment facilities [11].

The natural reservoirs of mammalian arenaviruses are specific rodent species, in which the viruses are most often maintained through a largely asymptomatic, persistent infection. Among rodents, arenaviruses are reported to be transmitted by both horizontal and congenital routes [12]–[14]. The geographic range of each arenavirus is typically thought to be determined by the range of its reservoir, and humans are most often infected through contact with excreta from these rodents [12]. TCRV is an arenavirus for which a rodent host has never been specified. Instead, it had been isolated from two species of *Artibeus* fruit bats in Trinidad from 1956 to 1958 during a rabies surveillance survey. It was also isolated from a single pool of 344 mosquitoes in 1956. For ten years following the initial isolation from mosquitoes, over 1 million mosquitoes of various species from Trinidad tested negative for the virus [13]. Recent experimental evidence indicates that bats are not the natural reservoir host for TCRV following virus inoculation into the Jamaican fruit bat [14], and extensive efforts to identify the virus in other mammals, mostly rodents, have failed [13]. Although 19 TCRV isolates were obtained from fruit-eating bats in the 1950s, only one, TRVL-11573, remains, leaving much about the ecology and epidemiology of the virus unknown. All other isolates obtained from bats were accidentally destroyed. TRVL-11573 has been extensively passaged, and is the only laboratory strain in existence for animal models of infection and for genetic comparison. Here, we preliminarily analyzed the genome sequence of the Florida isolate of Tacaribe virus derived from a pool of 100 adult host-seeking ticks. In addition, we determined the prevalence of TCRV vRNA in host-seeking *Amblyomma americanum* (lone star ticks; LST) collected in North Central Florida.

## Methods

### Tick collection and processing

**Figure 1:**
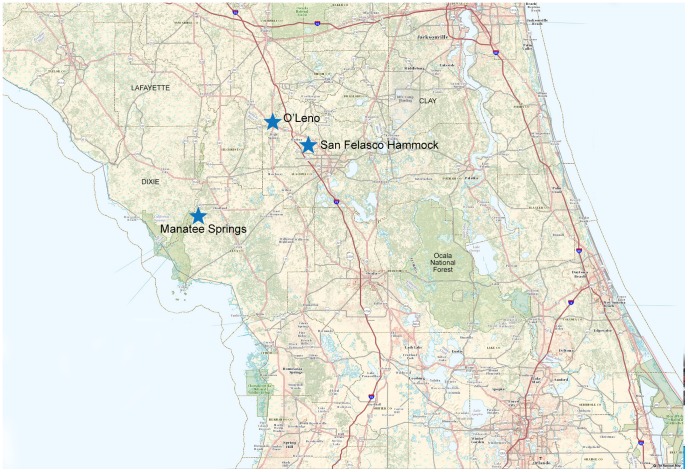
Field site locations in Central Florida, USA. Ticks were trapped for virus isolation at San Felasco Hammock State Preserve (center) in 2012. In addition to San Felasco Hammock, ticks were trapped at two additional sites in 2013 for screening by RT-qPCR for TCRV vRNA. Image produced with USGS National Map Viewer (http://viewer.nationalmap.gov/viewer/).

One hundred (100) adult host-seeking *A. americanum* were collected from San Felasco State Park, west of Gainesville, FL, USA, in March of 2012 (29°42′33.189″ N latitude and 82°27′23.213″ W longitude.). Tick trapping was performed using 1 m^2^ flannel drags in combination with carbon dioxide-baited traps as previously described [15]. All tick trapping was performed in accordance with the Florida Department of Environmental Protection Research and Collection Permit #05231210. Only host-seeking tick species common in Florida were collected because these species are most likely to attach to a person and take a blood meal. No protected or endangered species were sampled in this study. The ticks were identified according to standard taxonomic keys and immature ticks were removed from the sample pool. The sample pool was subsequently stored frozen at −80°C until homogenization for virus isolation. A 10% w/v tick homogenate was prepared for virus isolation as described in Sang et al. [16]. In 2013, ticks were collected using the same methods as 2012 from two additional Florida state parks: Manatee Springs State Park in Chiefland, Florida (29° 29′47.401″ N, 82°58′4.429″ W) and O'Leno State Park in High Springs, Florida (29°55′11.863″ N and 82° 35′15.427″ W), to determine if the virus could be detected in other locations in North Central Florida (Fig.1).

### Cell cultures

A variety of cell lines were tested to improve the chances of isolating mammalian viruses, as some viruses replicate in particular cell lines. Cell lines A549 (CCL-185), BHK-21 (CCL-10), HeLa (CCL-2), LLC-MK2 (CCL-7), MDCK, (CCL-34), Mv1 Lu (CCL-64), Neuro-2a (CCL-131), NIH/3 T3 (CRL-1658), and Vero E6 (CRL-1586) were obtained from the American Type Culture Collection (ATCC) (Manassas, VA, USA), and propagated as monolayers at 37°C and 5% CO_2_ in Dulbecco's Modified Eagle's Medium (DMEM) (Mediatech, Inc., Manassas, VA, USA) or Eagle's Minimal Essential Medium (EMEM) (Invitrogen, Carlsbad, CA, USA), as appropriate per cell line. DMEM and EMEM were supplemented with 2 mM L-Alanyl-L-Glutamine (GlutaMAX, Invitrogen, Carlsbad, CA, USA.), antibiotics [PSN; 50 µg/ml penicillin, 50 µg/ml streptomycin, 100 µg/ml neomycin (Invitrogen, Carlsbad, CA, USA)], and 10% (v/v) low IgG, heat-inactivated gamma-irradiated fetal bovine serum (HyClone, Logan, UT, USA). EMEM was also supplemented with sodium pyruvate (Invitrogen Corp.) and non-essential amino acids (Hyclone, Logan, UT, USA).

### Virus isolation

Cell lines were grown in 25-cm^2^ cell culture flasks to 80% confluency. After rinsing with cell growth media, the cells were inoculated with 0.5 ml of clarified tick homogenate prepared in DMEM with 10% serum and antibiotics. The inoculated cells were subsequently incubated at 37°C and observed daily for signs of cytopathic effects (CPE). The cultures were re-fed every 3 days, and blind-passaged every 2 weeks for a total of 6 blind passages before being considered negative for virus isolation. Non-infected cells were maintained and re-fed in parallel.

### Detection and Identification of TCRV RNA and virus particles

Impeded cell growth without overt CPE became apparent after the fourth blind-passage in both Neuro-2a and Vero E6 cell lines inoculated with tick homogenate, suggesting the presence of an infectious agent. In contrast, non-inoculated (negative control) Neuro-2a and Vero E6 cells replicated without signs of CPE. Multiple viral-based PCR primer sets were utilized for the detection of viral nucleic acids extracted from the spent media of both inoculated and non-inoculated Neuro-2a and Vero E6 cells (S1 Table). Amplicons were purified using a QIAquick PCR purification kit (Qiagen, Valencia, CA, USA), and plasmid clones of the amplicons were obtained using a TOPO-TA cloning kit according to the manufacturer's instructions (Life Technologies, Carlsbad, CA, USA). Plasmids from a total of 6 clones were bi-directionally sequenced at the University of Florida Interdisciplinary Center for Biotechnology Research. In parallel, electron microscopy was performed.

### Electron Microscopy

Inoculated Vero E6 cells were harvested, washed in 0.1 M phosphate buffer and pelleted by centrifugation at 2,500 rpm for 20 minutes. The supernatant was removed and the pellet resuspended in 2.5% gluteraldehyde in 0.1 M phosphate buffer and allowed to fix at 4°C overnight. Fixed cells were submitted to the Electron Microscopy Lab at the Malcolm Randall Veterans Affairs Medical Center, North Florida-South Georgia Veterans Health System, for processing and performance of transmission electron microscopy.

### Isolation of RNA from infected cell cultures

A QIAamp Viral RNA-mini kit (Qiagen) was used to extract RNA from liberated virions in the media of Vero E6 and Neuro-2a cells that had been inoculated with tick extract. Similarly, RNA was also extracted from Influenza B virus particles in the media of MDCK cells infected with that virus (non-template control), and from the spent media of non-inoculated cells of the same type grown in parallel under the same conditions (negative control). The RNA extracts were immediately stored at −80°C until cDNA could be generated using Accuscript High Fidelity 1^st^ strand cDNA kit (Agilent Technologies, Santa Clara, CA, USA) using both random 9-mers and TCRV-1 primer (see S2 Table for primer sequence).

### Genome sequencing

Complete genome sequencing was accomplished using primer-walking PCR of the L and S segments of the virus. Virus isolation and genome sequencing by RT-PCR took place in separate laboratories, in separate buildings, using different equipment. To generate overlapping PCR products of approximately 800 bp in length, 28 oligonucleotide primers were used: TCRV-1 through 18 for the L segment of the virus (S2 Table) and primers TCRV-19 through 28 for the S segment (S3 Table). RT-PCR was performed with 2.5 uL cDNA template in 50 uL reactions using Phusion High-Fidelity PCR master mix (New England Biolabs, Ipswich, MA, USA) per the manufacturer's instructions. To obtain the 5′ and 3′ ends of the viral L and S genomes, 5′ and 3′ systems for the Rapid Amplification of cDNA Ends (RACE) were used per the manufacturer's protocols (Life Technologies, Carlsbad, CA, USA). Nucleotide sequences from bidirectionally Sanger-sequenced amplicons were assembled in Sequencher DNA sequence analysis software v2.1 (Gene Codes, Ann Arbor, MI, USA). Complete contigs were translated into protein sequences using EMBOSS Transeq and Sixpack. Comparisons of the protein sequences of this isolate and TRVL-11573 (GenBank M20304 & J04340) were completed using ExPASy SIM tool.

### RNA isolation from ticks

Adult *A. americanum* were stored in molecular grade 70% ethanol at -80°C before they were processed for RNA extraction (Fisher Scientific, Hampton, NH, USA). On ice, individual ticks were submerged in 10% bleach solution for 10 minutes, followed by three washes with 70% molecular grade ethanol and a final wash with molecular grade water. Ticks were rapidly dissected on an ice-cold RNAase/DNAase free sterile Petri dish with sterile instruments (Genesee Scientific, San Diego, CA, USA). Salivary glands and midguts of adult ticks were removed and immediately placed into RNA*later* stabilization reagent (Qiagen). RNA was extracted from tick midguts and salivary glands using Quick-RNA MiniPrep (Zymo Research, Irvine, CA, USA). RNA was quantified by fluorometric quantitation (Qubit 2.0 Fluorometer, Life Technologies, Grand Island, NY, USA).

### RT-qPCR

Forward primer TCRV-1 (S2 Table) was used as described above to generate full-length viral cDNAs. When reactions were complete, cDNA samples were stored at −20°C until needed for RT-qPCR. A third designated laboratory was used for performing RT-qPCR; the third laboratory was separate from the laboratories in which the virus was isolated and separate from the laboratory in which nucleic acids were recovered from LSTs. TaqMan-based qPCR was performed with 5 uL of cDNA template in a total volume of 25 uL. The reactions were performed using the QuantiTect Probe PCR master mix (Qiagen) as follows: 95°C for 15 minutes, followed by 40 cycles of 95°C for 15 seconds, and 60°C for 60 seconds. The reaction mixture contained 600 µM of each primer and the probe at a concentration of 200 µM. TCRV-specific primers and FAM-labeled probe were designed to be highly specific for a 100 nucleotide region of the NP gene. A previously reported a TCRV-specific forward primer was used [14] with a TCRV-specific reverse primer designed for this study: 5′-TGACGACAGACCTGGAAGT-3′ and the dual-labeled TaqMan probe: 5′-FAM-CCAGCCATCACCTGACAAACACAG-TAMRA-3′. Standards were generated using ten-fold serial dilutions of N gene-recombinant plasmids. The assay sensitivity was determined using limited dilutions of cDNA generated from culture-isolated viral RNA spiked into a constant amount of tick genomic DNA from a virus negative tick. Assay results were analyzed using MJ Opticon Monitor software, version 3.1 (Bio-Rad Laboratories, Hercules, CA, USA). Molecular-grade water was used as a negative control.

## Results

**Figure 2:**
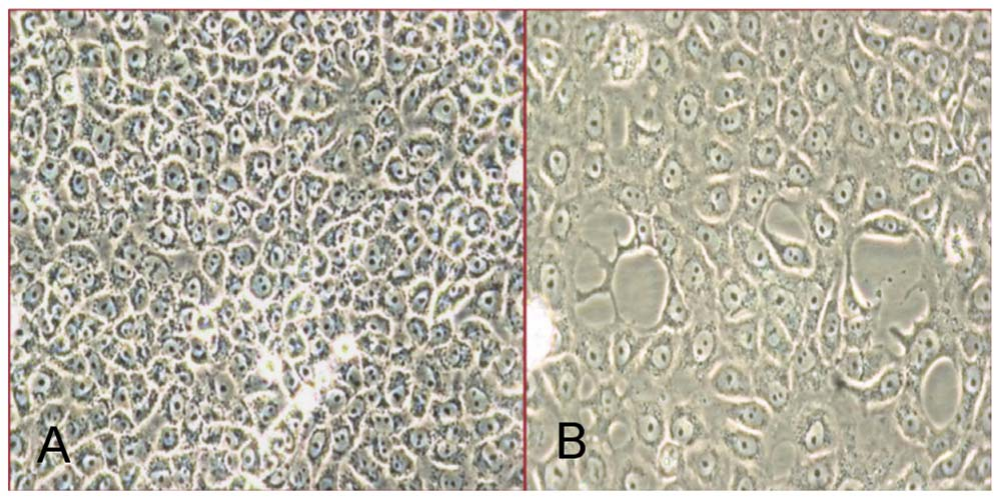
Non-infected Vero E6 control flasks (A) and Vero E6 flasks inoculated with tick homogenate (B) at day 13 post-inoculation.

**Figure 3:**
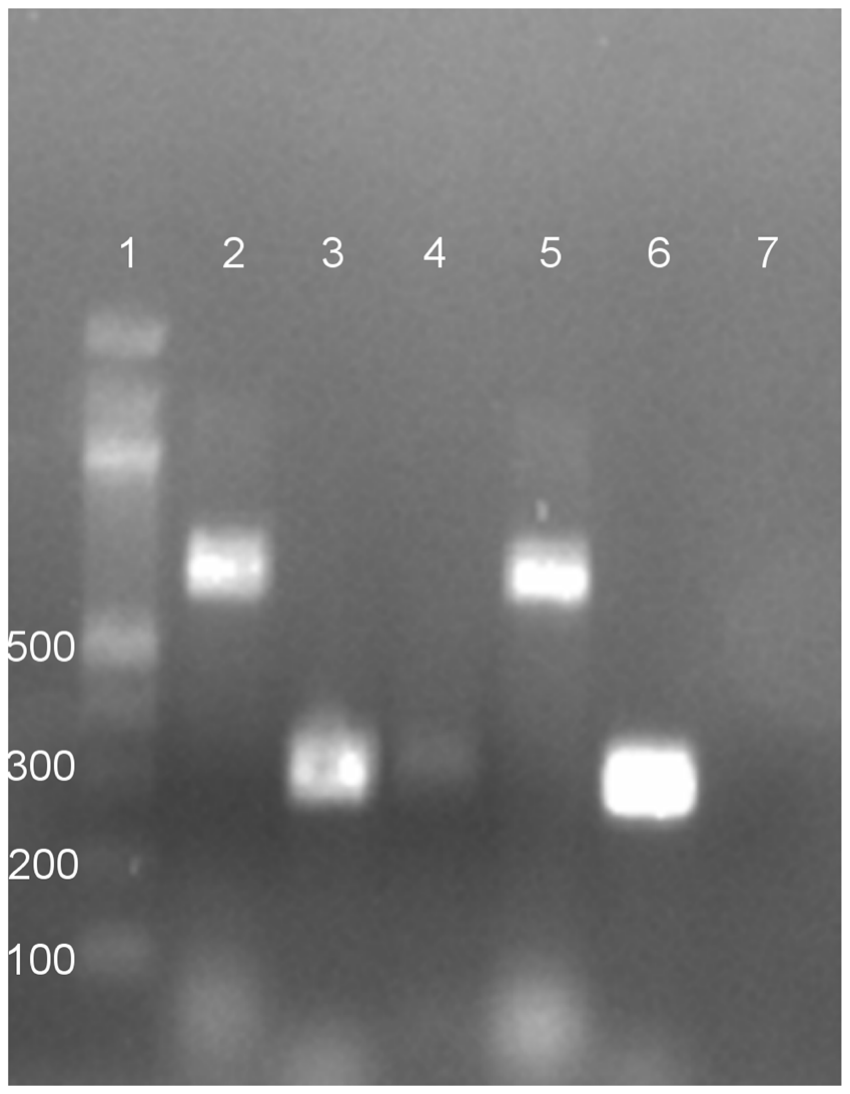
PCR products generated from Vero E6 cell supernates using pan-New World virus primers and Tacaribe primers. Bands in lanes 2 and 5 resulting in a 694 bp amplicon were generated using primers 1010C and 1696R as previously described by Bowen et al., 1997 [5]. Bands in lanes 3, 4 (light load) and 6 resulting in a 316 bp amplicon were generated using primers TACV-F and TACV-R as previously described by Cogswell-Hawkinson et al., 2012 [14]. Lane 1 contains a 100 bp ladder and lane 7 contains the non-template control. The gel was stained with ethidium bromide.

Impeded cell growth without overt CPE became apparent in both Neuro-2a and Vero E6 cell lines inoculated with tick homogenate after the fourth blind-passage. As noted by phase-contrast microscopy, the monolayer of inoculated flasks was subconfluent, whereas non-infected cells grew to confluency (Fig. 2). To gain clues on the identity of the infectious agent, universal primers specific for different DNA and RNA virus families were tested used in PCR or RT-PCR analyses. Among the family-specific primers, only RT-PCR primers capable of amplifying the North or South American arenaviruses produced amplicons from RNA extracted from virions in the spent media of infected Neuro-2a and Vero E6 cells. This indicated an arenavirus had been isolated. Subsequently, RT-PCR performed with TCRV-specific primers generated a product of the expected size. In contrast, no amplicons were formed from RNA that had been extracted from non-infected (negative control) cells (Fig. 3). Primers capable of amplifying the homologous region of LCMV-LASV viruses did not result in a product, ruling out the presence of any Old World viruses. To determine if a single species or quasi-species was present in infected cultures, both resultant amplicons visible in Fig. 3 were cloned. Basic local alignment search tool (BLAST) analyses of these clones revealed alignment to TCRV glycoprotein and nucleoprotein mRNAs (GenBank: M20304). There was 99% identity to 316 nucleotides and 98% identity to 694 nucleotides, to plasmid clones generated with TCRV-specific and pan-NWV primers, respectively.

**Figure 4:**
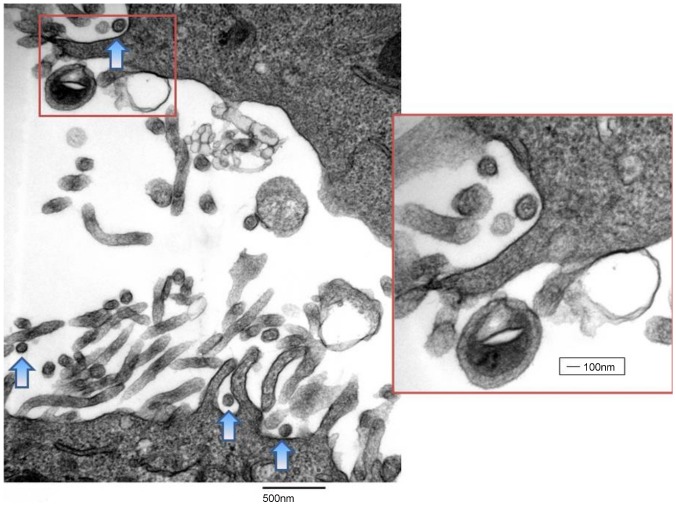
Transmission electron microscopy image of an arenavirus, identified by PCR as Tacaribe virus, in Vero E6 cells at magnifications of 33000x and 66000x (inset). Arrows indicate budding viruses that are emerging from the surface of a granulated Vero E6 cell.

Additionally, electron microscopy of fixed cells revealed the presence of virus particles with dimensions and features typical of arenaviruses (Fig. 4). Collectively, changes in the growth patterns of inoculated cell cultures, screening of the infected cells for viral RNA by RT-PCR, cloning and sequence analyses of amplicons, and electron microscopy revealed the presence of a NWV, specifically TCRV, had been isolated. The full genome sequence of virus isolate was derived by primer-walking and 5′ and 3′ RACE (described above, Methods), and the complete S and L segment sequences were submitted to GenBank (KF923400 and KF923401, respectively). The genome of our TCVR isolate had an overall identity of 99.3% to the prototype virus from Trinidad at the genome level. It was surprising to observe such a high level of conservation between two geographically and temporally distant isolates of the virus, as each of the four genes of the virus were 96.8-100% identical to TRVL-11573 isolate at the protein level (Table 1). Interestingly, the gap in the GPC that we observed due to the deletion of 12 amino acids at residues 123–134 in the published sequence [17] has been previously observed by other investigators [18] (S1 Fig.). Furthermore, our isolate deviated from the reference sequence at the four-amino acid region GPPT found at residues 389–392 of the NP gene. This deviation from the reference was previously reported by Harmon et al. in 2013 [1]. This group reported that the viral stocks obtained from the National Institutes of Health Biodefense and Emerging Infectious Diseases repository deviated from the reference sequence at this particular four-amino acid region, GPPT (GenBank KC329849) versus DLQL (GenBank NC004293), respectively, which was identical to the deviation that we report here (S2 Fig.). Furthermore, this group found that the TCRV NP gene had 98.2% identity to the reference published in GenBank, perhaps due to sequencing errors. Although errors may be present in sequences of TRVL-11573 accessioned in the early 1990's (M203304 and NC004293), the sequence of the Florida isolate reported here does deviate from any of the sequences reported previously in GenBank.

**Table 1:** Sequence identity of the Florida isolate of Tacaribe virus and the prototype virus.

Coding Region of Gene (Genbank accession number)	Nucleotide identity (%)	Amino acid identity (%)
NP (M20304)	1702/1713 (99.4)	562/570 (98.6)
GPC (M20304)	1415/1488 (95.1)	479/495 (96.8)
L (J04340)	6620/6633 (99.8)	2200/2210 (99.5)
Z (J04340)	288/288 (100)	95/95 (100)
S segment overall identity (coding regions)	3117/3234 (96.4)	1041/1065 (97.7)
L segment overall identity (coding regions)	7088/7102 (99.8)	2295/2305 (99.6)

**Figure 5:**
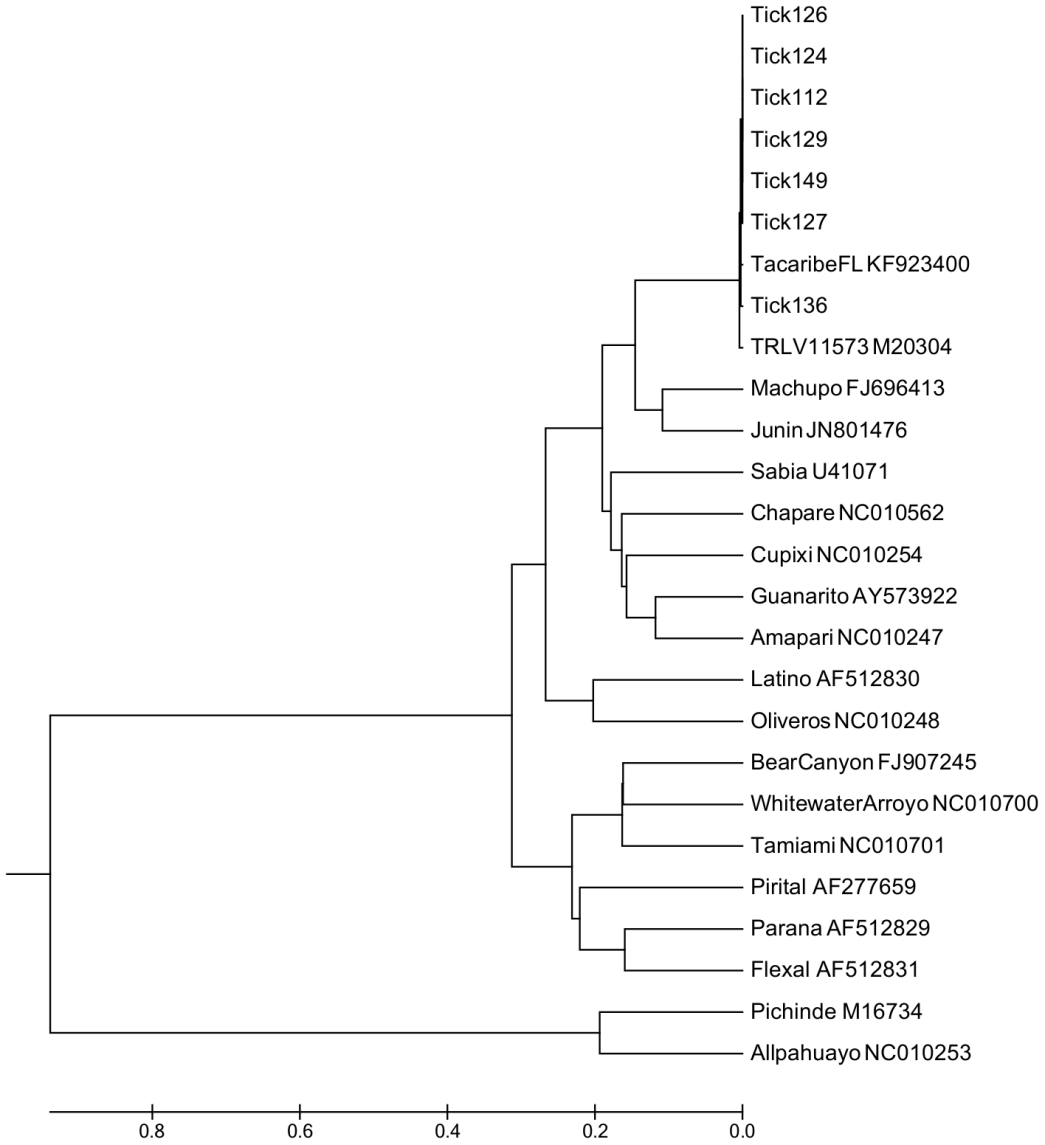
Arenavirus phylogenetic relationship inferred by MEGA software based on partial nucleotide sequences of the N gene of 18 NWVs and amplicons generated from PCR positive ticks. Arenaviruses are listed by name and immediately followed by the GenBank accession number of the strain used in this analysis. Numbers 112, 124, 126, 127, 129, 136 and 149 represent individual ticks with the greatest number of virus copies detected by RT-qPCR. The evolutionary history was inferred using the UPGMA method. The tree is drawn to scale, with branch lengths in the same units as those of the evolutionary distances used to infer the phylogenetic tree. The evolutionary distances were computed using the Maximum Composite Likelihood method and are in the units of the number of base substitutions per site.

Next, to provide further evidence of this arenavirus in the local *A. americanum* population, ticks were collected from February through July of 2013 and screened for the presence of vRNA using a RT-qPCR assay which targets the nucleocapsid gene of TCRV. A total of 500 host-seeking ticks were collected from three state parks located in North Central Florida, including the original field site where ticks were trapped for virus isolation attempts (Fig. 1). Collection site and pool size tested from each park was based on host-seeking tick availability. TCRV vRNA was detected in 0–25.1% of the ticks, depending on the site (Table 2). Overall, a total of 56/500 ticks (11.2%) had detectable quantities of TCRV vRNA. RT-qPCR positive ticks with the highest viral load were selected for conventional RT-PCR of a longer target and directly sequenced as described above. PCR products generated directly from ticks were used for genetic analysis. Amplicons generated from TCRV RNA positive ticks were nearly identical, providing further evidence of the presence of the virus in ticks in north central Florida (Fig. 5). This finding indicates that the virus was persistent in the local environment over a two year period.

**Table 2:** Prevalence of Tacaribe vRNA detected in host-seeking *A. americanum* by RT-qPCR.

Date of collection	Florida state park collection site	Number of positive ticks (%)
2013 Feb 28	O'Leno	0/19 (0%)
2013 May 25	Manatee Springs	42/167 (25.1%)
2013 May 28	San Felasco	6/88 (6.82%)
2013 Jun 14	Manatee Springs	4/48 (10.4%)
2013 Jun 26	San Felasco	4/178 (2.25%)
Overall prevalence:		56/500 (11.2%)

## Discussion

During this investigation, TCRV was detected in host seeking *A. americanum* ticks, as evidenced by isolation of the virus in cell cultures, and RT-qPCR detection of viral RNA in RNA extracted from bleached and individually dissected ticks. We fully sequenced the genome of this isolate by primer walking and bidirectional sequencing of resultant amplicons. The isolation of this virus from host-seeking ticks in San Felasco Hammock State Preserve in 2012 was unexpected. In 2013, we again detected TCRV vRNA in ticks from San Felasco and also at a second state park. The latter findings indicate that the virus was maintained from 2012 to 2013 in the tick subpopulation in San Felasco and was also present in ticks collected from another park approximately 80 km away.

Our awareness of emerging tick-associated viruses is increasing. Recently, bunyaviruses, particularly those in the genus *Phlebovirus*, have been associated with tick-borne illnesses in the United States, Eurasia, Africa and China [19]–[20]. In 2011, a novel phlebovirus vectored by the hard tick, *Haemaphysalis longicornis*, was documented to be the etiological agent of Severe Fever with Thrombocytopenia Syndrome Virus (SFTSV) in the Hubei and Henan provinces of China [20]. Additionally, recent reports of hospitalization caused by Heartland virus in the central United States, has increased public health awareness of tick-borne phleboviruses [21]. However, arenaviruses have not previously been reported as causes of tick-associated illnesses, although fatal illnesses have been associated with a NWV in California [22] and recently a novel arenavirus infection was documented in the United States [23]. The possibility of vector transmission of this, and perhaps other arenaviruses, needs to be further evaluated.

The Florida isolate of TCRV shares over 98% amino acid similarity with the original isolate (NC004292, NC004893), although the latter was isolated nearly 60 years ago and the mutation rates of single-stranded RNA viruses are known to be high. Virus diversity can be greatest near the source of origin, [24] and although we have propagated the virus from a single geographic location in Florida, a quantitative RT-PCR targeting the nucleocapsid gene of TCRV detected gene copies in host-seeking ticks from multiple locations. Sequencing of longer nucleocapsid transcripts of approximately 300 bp amplicons from RT-qPCR positive ticks revealed no genetic variability in the 5′ end of this gene of the virus circulating in ticks. The nucleocapsid gene of the virus was selected because it is the most abundant target in circulating virus, it is involved with virus packaging, and early studies with arenaviruses indicated this protein is an antigenic site and therefore presumably under selective pressure [25]. Sequences amplified from ticks were highly conserved, indicating perhaps that the population of the virus in the potential vector here may be at equilibrium and that unfavorable genotypes have been eliminated. However, longer transcripts would have to be amplified from ticks and analyzed to determine whether or not this is a reasonable conclusion. Future studies should isolate the virus from more geographically disparate locations through Florida and beyond, as the frequency at which alternative TCRV sequences circulate in nature is currently unknown. Having multiple isolates is crucial, because although nearly twenty TCRV isolates were originally obtained from bats in Trinidad in the 1950s [13], only strain TRVL-11573 remains available for analysis and comparison. This strain has undergone 24 passages in suckling mice and 2 passages in Vero E6 cells [14] and sequence variation has been reported by multiple investigators despite the fact that all viral stocks of TCRV have been derived from isolate TRVL-11573 [11], [18]. Proposed reasons for these differences are the use of different methods of amplification or sequencing, or due to duration of passaging in cell culture. It is important to know how the pathogenicity of TCRV isolates change in culture over time because some arenaviruses, such as Pichinde virus, become more pathogenic after sustained serial passages [26], whereas other arenaviruses lose their pathogenicity [27]. Having multiple isolates would be helpful for understanding the evolution of this virus across time, space, and perhaps between reservoirs. Furthermore, it is possible to develop serological assays using the NP protein to screen mammalian reservoirs in Florida, particularly in locations where we know the virus is circulating in ticks. Determining the reservoir and range of the virus, as well as the role of *A. americanum* in the ecology of the virus, is essential for determining the population dynamics of the virus. In future work, the ability of the tick to transmit the virus needs to be evaluated in order to delineate the risk of human infection following a tick bite.

Although this finding is novel and arenaviruses have not been associated with ticks historically, we are confident that this finding is not a contaminant. Molecular evidence that the virus in Florida is not a contaminant is: 1) The investigators have never worked with Tacaribe virus previously. It has never been handled in or near the facilities used by the investigators. The work performed in the laboratories where the full genome sequencing, RNA isolation from ticks, and RT-qPCR took place has previously focused on obligate, intracellular tick-borne bacteria, particularly *Anaplasma* and *Ehrlichia* spp.; 2) TCRV grew in inoculated cells but not controls, as evidenced by impeded growth in cells inoculated with tick homogenate but not in controls maintained under the same conditions; 3) RNA extracted the contents of the spent medium of infected and non-infected cells was subjected to RT-PCR using a variety of primers. Amplicons were generated using two primer sets capable of amplifying TCRV RNA (NWV primers and TCRV-specific primers), and those primers failed to amplify a product from mock-infected or non-infected controls; 4) Cloning of amplicons, direct sequencing and electron microscopy performed in parallel confirmed the presence of an arenavirus, specifically TCRV, in inoculated cells but not in controls; 5) In a separate laboratory, twenty-four new, previously unhandled primers were used to sequence the genome of the virus. Negative controls (molecular grade water) failed to produce any bands in any of these reactions, whereas cDNA generated using either random 9-mers or TCRV-specific primers (TCRV-1), produced identical bands, indicating that our oligonucleotides were not a source of contamination; 6) Reagents purchased from two different manufacturers were used to recover nucleic acids from the contents of spent medium in 2012 and from ticks in 2013 (Qiagen versus Zymo, respectively), suggesting that these reagents are not a source of TCRV vRNA; 7) a different set of primers and a TCRV-specific probe was used to provide molecular evidence of viral RNA in ticks by RT-qPCR, which was performed in a separate laboratory. Negative controls (molecular grade water) included on every plate did not generate a signal, nor did negative ticks previously screened by RT-PCR for TCRV vRNA. Samples were run in duplicate and RT-qPCR positive ticks were subjected to another PCR in order to generate amplicons of adequate length for phylogenetic analysis. The use of controls and replicates used for this assay demonstrate the reliability of the assay.

In conclusion, we isolated TCRV, a Caribbean arenavirus, in mammalian cells from a homogenate of 100 host-seeking adult *A. americanum* collected in north central Florida. We fully sequenced the genome of this isolate and detected TCRV vRNA in ticks from the same location, and in an additional location the following year.

## Supporting Information

S1 Fig **Tacaribe glycoprotein (GPC) sequence alignment.** Reference sequence (NC004293) compared with the Florida isolate (KF923400). Differences are highlighted in yellow.
(TIF)

S2 Fig **Tacaribe nucleoprotein (NP) sequence alignment.** Reference sequence (NC004293) compared with the Florida isolate (KF923400). Differences are highlighted in yellow.
(TIF)

S1 Table **Primers used to identify the infecting arenavirus in culture**.
(DOCX)

S2 Table **Primers designed for walking the large (L) segment.**
(DOCX)

S3 Table **Primers designed for walking the short (S) segment.**
(DOCX)
