# Supplementary figures and images for: Isolation of Tacaribe Virus, a Caribbean Arenavirus, from Host-Seeking Amblyomma americanum Ticks in Florida
Source: PLoS One. 2014 Dec 23;9(12):e115769. doi: 10.1371/journal.pone.0115769 (PMC4275251; doi:10.1371/journal.pone.0115769)

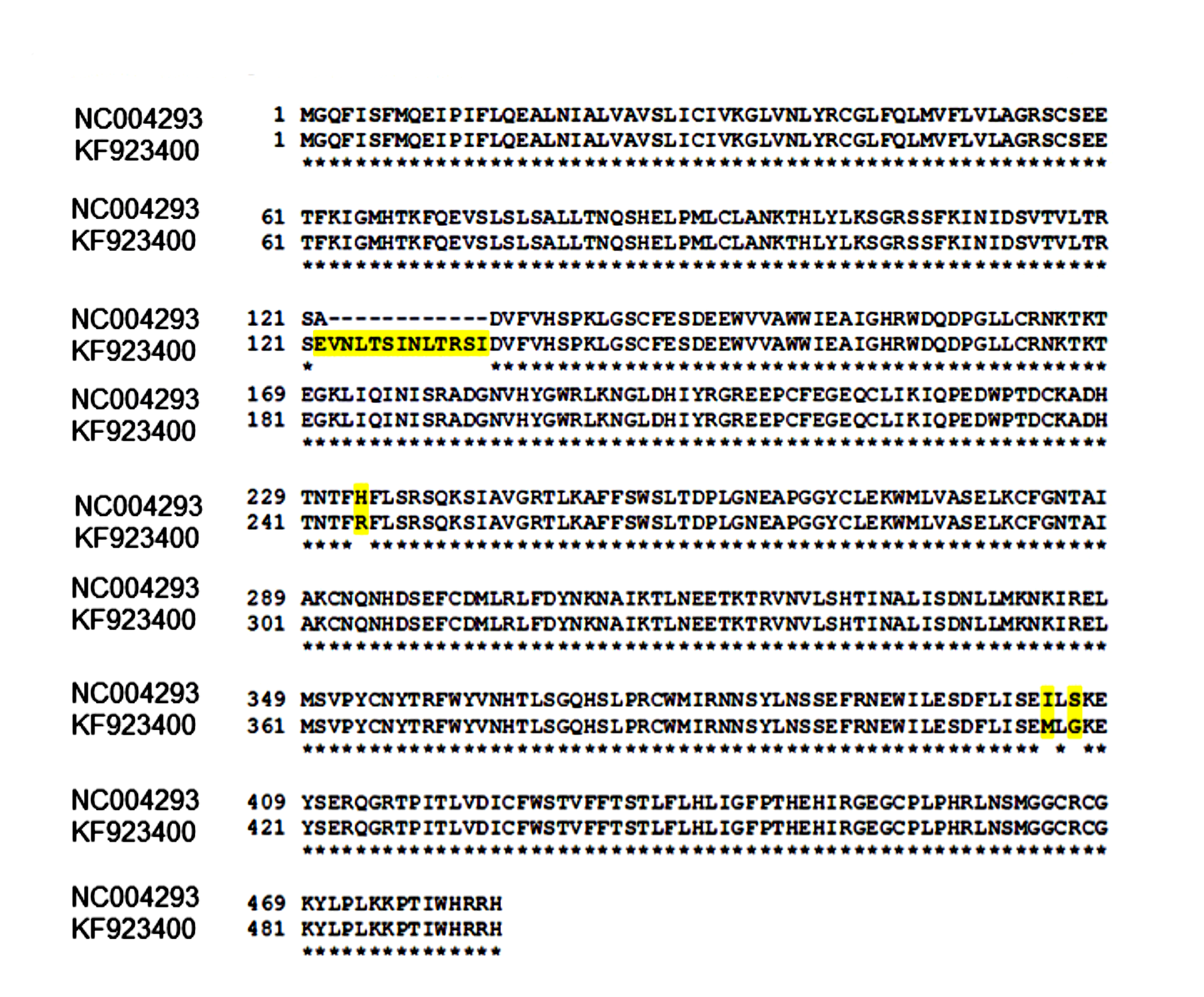

Supplement: S1 Fig — Tacaribe glycoprotein (GPC) sequence alignment. Reference sequence (NC004293) compared with the Florida isolate (KF923400). Differences are highlighted in yellow. (TIF) [file pone.0115769.s001.tif]

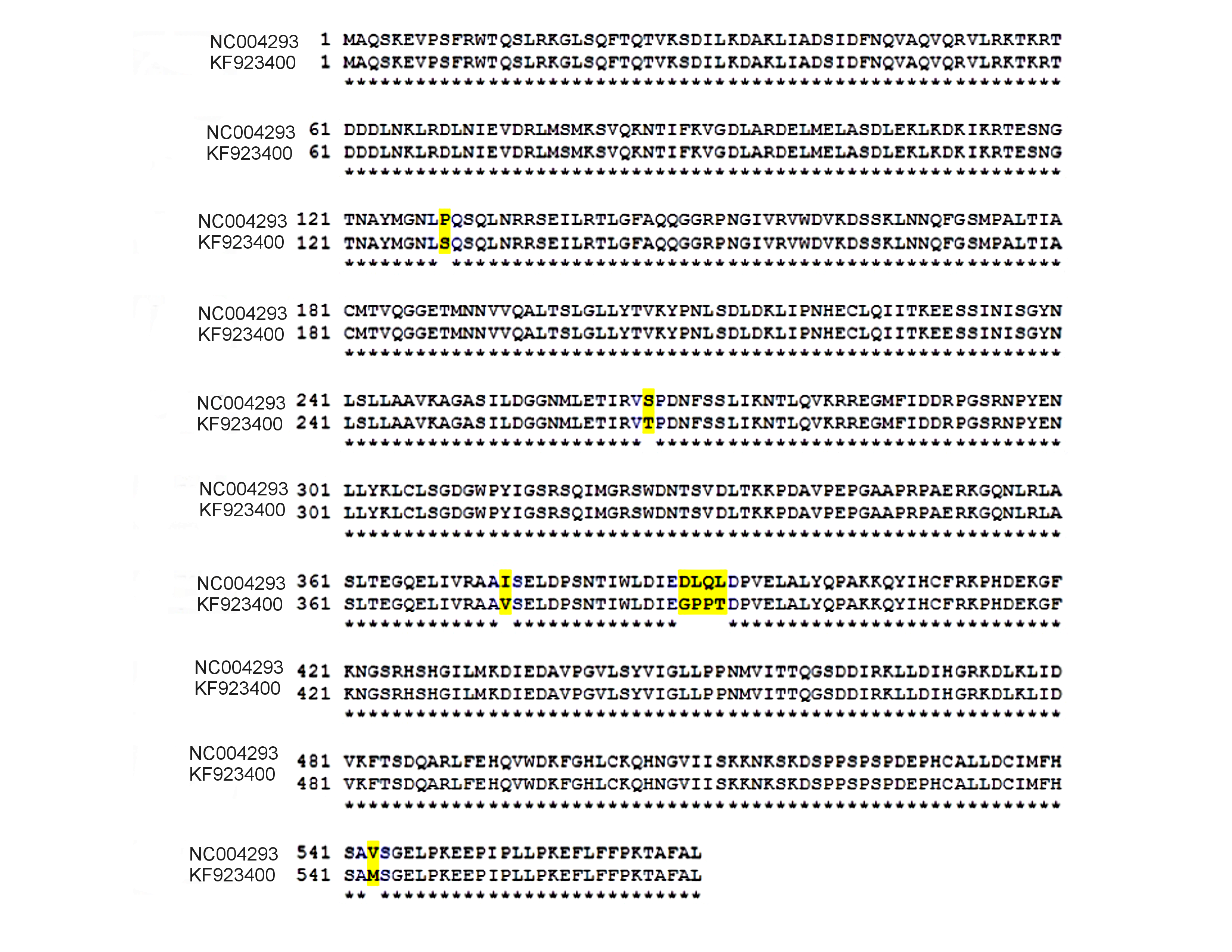

Supplement: S2 Fig — Tacaribe nucleoprotein (NP) sequence alignment. Reference sequence (NC004293) compared with the Florida isolate (KF923400). Differences are highlighted in yellow. (TIF) [file pone.0115769.s002.tif]
