# Supplementary material for: Isolation of Tacaribe Virus, a Caribbean Arenavirus, from Host-Seeking Amblyomma americanum Ticks in Florida
Source: PLoS One. 2014 Dec 23;9(12):e115769. doi: 10.1371/journal.pone.0115769 (PMC4275251; doi:10.1371/journal.pone.0115769)
Supplement: S1 Table — Primers used to identify the infecting arenavirus in culture. (DOCX) [file pone.0115769.s003.docx]

Table S1. Arenavirus primers used to identify the infecting virus in culture

| Target | Primer name | Sequence | Reference |
| --- | --- | --- | --- |
| N gene complementary sequence | 1010C | TCIGGIGAIGGITGGCC | Bowen, 1996 |
| N gene Tacaribe and MOP viruses | NW1696R | ACATIAIGIIGTCIAGIAGIGCACAGTG | Bowen, 1996 |
| N gene of all LCMV-LASV | OW1696R | AIATGAIGCAGTCCAIIAGTGCACAGTG | Bowen, 1996 |
| Tacaribe virus specific | TACV forward | AATTTGCGATCGAGAGCCTA | Cogswell-Hawkinson, 2012 |
|  | TACV reverse | AGCTCATCCCAAACCATGAG | Cogswell-Hawkinson, 2012 |
