# Supplementary material for: Isolation of Tacaribe Virus, a Caribbean Arenavirus, from Host-Seeking Amblyomma americanum Ticks in Florida
Source: PLoS One. 2014 Dec 23;9(12):e115769. doi: 10.1371/journal.pone.0115769 (PMC4275251; doi:10.1371/journal.pone.0115769)
Supplement: S2 Table — Primers designed for walking the large (L) segment. (DOCX) [file pone.0115769.s004.docx]

Table S2. Primers designed for walking the large (L) segment

| Primer | Base position | Length | GC content (%) | Tm in °C (salt-adjusted) | Sequence |
| --- | --- | --- | --- | --- | --- |
| TCRV-1 | 816 | 20 | 50 | 58.4 | AAG TGG CCG TCT CTT AAC AC |
| TCRV-2 | 1600 | 22 | 41 | 58.4 | TAG ATA AGG AAG TTG GAC TGG A |
| TCRV-3 | 1523 | 23 | 40 | 59.2 | AGT TGA CAT CAC TAA AGG GTT CA |
| TCRV-4 | 2316 | 20 | 50 | 58.4 | TCT GTA ACC AAC GAC AAA CAA G |
| TCRV-5 | 2254 | 23 | 39 | 59.2 | ATT TGT TTA TGG ACT CCG CTA AG |
| TCRV-6 | 3061 | 22 | 41 | 58.4 | CCT TGT GCT TCA GAT GAT AAT C |
| TCRV-7 | 2947 | 17 | 55 | 58.4 | TGC CCG CTA CAC TCT TG |
| TCRV-8 | 3741 | 22 | 41 | 58.4 | CTG ATG ATT CTC CTA CTG AGT T |
| TCRV-9 | 3675 | 22 | 41 | 58.4 | ACG TAA AGT TCC CTA TTG GAA C |
| TCRV-10 | 4425 | 20 | 50 | 58.4 | CCA GAG AGA TTG TGA AGA GC |
| TCRV-11 | 4279 | 21 | 43 | 57.5 | TCG GAC TTA GAT TGA CAT CTC |
| TCRV-12 | 5065 | 19 | 58 | 59.5 | CTG GCA GTG GAC CTT TTC C |
| TCRV-13 | 4984 | 22 | 45 | 60.1 | AGA GAT ACT TGA ACC TCC TAG C |
| TCRV-14 | 5643 | 21 | 48 | 59.5 | TGA TGT CAG ATG CTG AAC AGG |
| TCRV-15 | 5615 | 20 | 50 | 58.4 | AGC CTC AAC CTG TTC AGC AT |
| TCRV-16 | 6389 | 23 | 45 | 56.4 | CGA TGT TCG AGA ATC TGT GA |
| TCRV-17 | 6217 | 20 | 50 | 58.4 | AAG GGA CAA TCT GAG GTG GT |
| TCRV-18 | 7044 | 21 | 48 | 59.5 | CCA TGG ATG AAA CTG TGT CTG |
