# Supplementary material for: Isolation of Tacaribe Virus, a Caribbean Arenavirus, from Host-Seeking Amblyomma americanum Ticks in Florida
Source: PLoS One. 2014 Dec 23;9(12):e115769. doi: 10.1371/journal.pone.0115769 (PMC4275251; doi:10.1371/journal.pone.0115769)
Supplement: S3 Table — Primers designed for walking the short (S) segment. (DOCX) [file pone.0115769.s005.docx]

Table S3. Primers designed for walking the short (S) segment

| Primer | Base position | | Length | GC content (%) | Tm in °C (salt-adjusted) | Sequence |
| --- | --- | --- | --- | --- | --- | --- |
| TCRV-19 | | 168 | 20 | 50 | 58.4 | CAG GTG CGG ACT GTT TCA AT |
| TCRV-20 | | 754 | 18 | 61 | 58.4 | CAG TCG GCC AGT CTT CAG |
| TCRV-21 | | 619 | 22 | 41 | 58.4 | TTC AAG AGC TGA TGG CAA TGT T |
| TCRV-22 | | 1438 | 20 | 50 | 58.4 | TCC CCT CGA ATG TGT TCA TG |
| TCRV-23 | | 1195 | 20 | 50 | 58.4 | CAC TCT CTT CCA AGA TGC TG |
| TCRV-24 | | 1946 | 22 | 41 | 58.4 | CAC GGA AGG AAA GAT CTC AAA T |
| TCRV-25 | | 1872 | 20 | 50 | 58.4 | CAC TTG GTG CTC GAA TAG TC |
| TCRV-26 | | 2616 | 20 | 50 | 58.4 | CAG TCT GTT GGC AGC AGT AA |
| TCRV-27 | | 2573 | 21 | 43 | 58.4 | TCT CCA ACA TGT TAC CAC CAT |
| TCRV-28 | | 3374 | 21 | 43 | 57.5 | CTT GCT TTG ATC GCC ATA ATG |
